# Effectiveness of Treatment Modalities for the Correction of Anterior Crossbite in Children: A Systematic Review and Meta-Analysis of Randomized Controlled Trials

**DOI:** 10.7759/cureus.110226

**Published:** 2026-06-04

**Authors:** Yazan M Kourbaj, Mohammad Y Hajeer, Ahmad S Burhan, Mohammad Khursheed Alam, Huda Abutayyem

**Affiliations:** 1 Department of Orthodontics, Faculty of Dentistry, University of Damascus, Damascus, SYR; 2 Department of Preventive Dentistry, College of Dentistry, Jouf University, Sakaka, SAU; 3 Department of Clinical Sciences, Center of Medical and Bio-Allied Health Sciences Research, College of Dentistry, Ajman University, Ajman, ARE

**Keywords:** anterior crossbite, class iii malocclusion, correction of incisor inclination, crossbite with displacement, early orthodontic treatment, mixed dentition, orthodontic treatment, reverse overjet

## Abstract

Anterior crossbite in children is a common malocclusion that, if left untreated, may lead to occlusal dysfunction, impaired craniofacial growth, and long-term periodontal or temporomandibular complications; however, a comprehensive synthesis of high-quality evidence comparing the effectiveness of different early treatment modalities across skeletal, functional, and dental etiologies remains limited.

This study was designed as a systematic review and meta-analysis to investigate and compare the efficacy of different orthodontic approaches for treating anterior crossbites in children during their growth phase. A thorough search of the literature was performed up to September 2025 across several databases, including PubMed, Cochrane Library, Scopus, Web of Science, and Google Scholar, without applying any restrictions on language or publication year. Only randomized controlled clinical trials (RCTs) focusing on children aged 6-12 years who received orthodontic treatment for anterior crossbite correction were considered eligible. Two reviewers independently handled the processes of study selection, data collection, and risk-of-bias evaluation using the RoB-2 tool, with any disagreements resolved through consultation with a third reviewer. Data synthesis was carried out using RevMan 5.4, and the strength of the evidence was assessed according to the GRADE guidelines.

A total of 21 RCTs involving 854 participants were included, comprising 18 studies on skeletal, two on functional, and one on dental anterior crossbite. Intraoral non-skeletally anchored appliances demonstrated modest improvements in sagittal skeletal relationships (ANB mean difference (MD) = 0.29°-3.12°) and clinically meaningful overjet correction (MD = 1.4-5.9 mm), primarily through dentoalveolar mechanisms rather than true skeletal modification. In contrast, facemask therapy combined with rapid maxillary expansion (FM-RME) produced the most pronounced skeletal effects, with pooled estimates showing significant increases in ANB (MD = 3.54°) and SNA (MD = 1.37°), along with a reduction in SNB (MD = -2.14°), although substantial heterogeneity was observed across studies. Skeletally anchored facemask therapy did not demonstrate a clinically meaningful advantage in overall sagittal correction compared with conventional tooth-borne facemask therapy (ANB MD = 0.07°), although a small additional improvement in maxillary advancement was noted (SNA MD = 0.59°). Considerable variability existed across trials in terms of appliance design, treatment protocols, follow-up duration, and outcome assessment methods. The overall certainty of evidence ranged from very low to moderate.

In conclusion, early orthodontic treatment of anterior crossbite in growing children is effective, with outcomes largely influenced by the underlying etiology and the type of appliance used. Intraoral appliances are appropriate for dental and functional crossbites and mild skeletal discrepancies, whereas FM-RME remains the most reliable modality for achieving significant skeletal correction in true skeletal anterior crossbite cases. The additional benefit of skeletal anchorage appears to be limited compared with conventional approaches. Further high-quality, standardized RCTs with consistent outcome measures and long-term follow-up are needed to increase the evidence base strength, because of the heterogeneity of included studies and the typically low confidence of the evidence.

## Introduction and background

Anterior crossbite is a sagittal discrepancy in which the maxillary incisors are positioned on the lingual side of the mandibular incisors, which can hinder normal maxillary development and necessitate early orthodontic intervention [1]. This misalignment restricts lateral mandibular movements, potentially leading to functional limitations and occlusal imbalances [2]. Prolonged anterior crossbite is associated with significant incisal wear, which may affect either the maxillary or mandibular incisors, increasing the risk of dental complications over time [2]. Additionally, continuous occlusal trauma can affect the stability and long-term prognosis of the incisors [3], while repeated anterior displacement of the mandibular condyle may disrupt the condyle-joint relationship, potentially leading to temporomandibular joint dysfunction [3]. Gingival recession is another concern, particularly for the lower incisors, as poor oral hygiene and gingival inflammation can accelerate periodontal disease [2].

Anterior crossbite is categorized into three types based on its underlying causes. Dental crossbite occurs due to factors such as retained primary teeth, the presence of supernumerary teeth, reduced upper arch length, incorrect initial positioning of incisor buds, trauma to maxillary primary teeth that reorient the permanent tooth buds lingually, or habits like upper lip biting [2]. Functional crossbite is caused by an early occlusal contact point, leading to forward displacement of the lower jaw, resulting in a pseudo Class III malocclusion [4]. Skeletal crossbite arises from maxillary retrusion, mandibular protrusion, or a combination of both, often influenced by genetic factors affecting jaw position and size [5].

Early anterior crossbite treatment aims to prevent progressive irreversible soft tissue or hard tissue changes, improve skeletal discrepancies and provide a more favorable environment for future growth, improve occlusal function, reduce the need for orthognathic surgery, and provide more aesthetically pleasing facial features, all of which improve the psychosocial development of a child [6].

Several approaches have been employed to manage dental and functional Class III malocclusion in mixed dentition. These include techniques such as the tongue blade, removable appliances with an inclined plane, and removable appliances with push springs, all designed to reposition anterior teeth [6]. Additionally, fixed orthodontic devices have been utilized to provide continuous corrective forces [6]. On the other hand, skeletal Class III malocclusion requires interventions that modify growth patterns. Growth modification strategies incorporate a range of orthopedic and functional appliances, including the face mask [7], chin cup [8], the removable mandibular retractor (RMR) [9], Bionator III [10], reversed twin block [11], Frankel III regulator [12], and the modified tandem appliance [13].

While reviewing the literature, several systematic reviews have been published that evaluate the evidence on the effectiveness of available tools for correcting anterior crossbite in growing patients [14,15]. Khalaf and Mando conducted a systematic review to assess the effectiveness of removable appliances in correcting anterior dental and functional crossbite [14]. While their findings suggest that these appliances can be beneficial, the strength of the evidence was classified as moderate to very low according to the GRADE framework [14]. Additionally, the review included only three randomized controlled trials (RCTs), which limits the strength of its conclusions. As a result, the authors suggested that further high-quality RCTs are needed to provide stronger evidence for the effectiveness of removable appliances in treating anterior crossbite [14].

Similarly, Jorge et al. compared fixed and removable appliances for the early treatment of non-skeletal anterior crossbite [15]. Their findings indicated that fixed appliances were associated with shorter treatment duration and lower costs compared with removable devices. However, according to the GRADE framework, the evidence supporting overjet correction was considered to be of very low quality [15]. The most recent systematic review in this regard examined various orthodontic devices used to correct skeletal Class III malocclusion [16]. The review focused on patients aged 16 years or younger, an age generally considered too young for such interventions [16].

To our knowledge, there are currently no systematic reviews examining early intervention strategies for anterior crossbites in children across skeletal, functional, and dental etiologies. The existing literature does not provide a comprehensive, integrated assessment of treatment approaches that address all three domains collectively, indicating a gap in evidence-based recommendations for the early management of anterior crossbite in orthodontics. Accordingly, this systematic review was performed to address the following targeted review question: “What is the most effective treatment method for the management of skeletal, functional, and dental anterior crossbites in children?”

## Review

Materials and methods

Preliminary Search and Protocol Registration

An initial pilot search was conducted in PubMed to develop the protocol for this systematic review, to confirm that no similar reviews existed, and to identify pertinent studies. The protocol was then registered in PROSPERO at an early stage of the study (CRD420251171927). This systematic review was prepared in accordance with the Cochrane Handbook for Systematic Reviews of Interventions, along with the relevant checklist [17], and followed the Preferred Reporting Items for Systematic Reviews and Meta-Analyses (PRISMA) guidelines [18,19].

Eligibility Criteria

The eligibility criteria for this systematic review were established using the PICOS framework, which defines the Population, Intervention, Comparison, Outcomes, and Study design. The population included growing children aged between 6 and 12 years, of any gender or ethnic background, who had an anterior crossbite. The intervention of interest was any orthodontic approach used to correct an anterior crossbite. The comparison group consisted of individuals who either received no orthodontic treatment or were treated with a different type of appliance than that used in the intervention group. Outcomes of interest included changes in skeletal relationships, overjet, and overbite. Only RCTs were eligible for inclusion, with no language or publication date limitations.

The following types of studies were excluded: case reports, case series, non-randomized clinical trials (CCTs), opinion articles, editorials, systematic reviews, studies lacking a defined sample size, and studies in which the experimental group comprised fewer than 10 patients.

Search Strategy

Two independent reviewers (YMK and MYH) carried out a comprehensive electronic search in September 2025. The databases searched included PubMed, the Cochrane Central Register of Controlled Trials (CENTRAL), Scopus, Web of Science, and Google Scholar. No restrictions were applied regarding language or publication period. The search terms used are presented in Table 1, while a detailed description of the search strategy is provided in Appendix 1. In addition, the reference lists of all included studies and relevant review articles were manually screened to identify any additional eligible studies that may not have been captured through the electronic search process.

**Table 1:** Keywords used in the electronic search.

Component of the search strategy	Keywords and the related terms
Type of malocclusion	Class III malocclusion, skeletal Class III, mandibular prognathism, maxillary retrusion, mandibular protrusion, skeletal anterior crossbite, functional anterior crossbite, dental anterior crossbite, mixed dentition.
Treatment planning	Growth modification, functional treatment, functional orthopedics, jaw relationship correction, maxillary advancement, mandibular restriction, upper incisor proclination, lower incisor retroclination, labial tipping of upper incisor, lingual tipping of lower incisor.
Outcomes	Skeletal improvement, overjet, overbite, upper incisor inclination, lower incisor inclination, facial profile, facial convexity, nasolabial angle, mentolabial angle, upper lip position, lower lip position.
Intervention	Removable orthodontic appliance, functional appliance, facemask, chin cup, Class III Activator, Class III Bionator, reverse pull headgear, Frankel III, reverse twin block (RTB), removable mandibular retractor (RMR), tandem appliance, modified tandem appliance, lower-clear-plate-based intermaxillary traction (LCP-IMT), bone-anchored maxillary protraction (BAMP), bone-anchored inter-maxillary traction (BAIMT)

Study Selection and Data Extraction

Two reviewers (YMK and MYH) evaluated the identified studies to assess their eligibility for inclusion in the review. Any disagreements were addressed through discussion with a third reviewer (MKA). The selection process began with screening of titles and abstracts, followed by full-text assessment of the potentially relevant articles. Studies that did not satisfy at least one of the inclusion criteria were excluded from the review. In addition, YMK and MYH independently performed data extraction from the included studies using standardized data collection forms. In cases of discrepancy, a third reviewer (MKA) was consulted to reach consensus. The extracted information included general study details, such as authors and year of publication, study design, sample size, participants’ mean age, type of malocclusion, intervention characteristics, duration of follow-up, treatment period, and reported outcomes.

Risk of Bias of the Collected Studies

The studies were assessed for potential bias using the Cochrane Risk of Bias (RoB 2.0) tool [20]. Two authors (YMK and MYH) independently assessed the risk of bias in each trial and compared their assessments. If there was a disagreement, a third reviewer (MKA) was consulted to reach a consensus. The risk-of-bias assessment focused on five domains: the randomization procedure (selection bias), adherence to the intended interventions, completeness of outcome data (attrition bias), methods of outcome measurement, and selective reporting of results. Overall bias was judged using predefined criteria. A study was considered to have a low risk of bias when all domains were rated as low risk. If at least one domain raised some concerns but none were at high risk, the study was classified as having “some concerns.” A high risk of bias was assigned when one or more domains were judged to be at high risk, or when multiple concerns across domains were significant enough to compromise confidence in the study outcomes. The researchers used the Risk of Bias Visualization (ROBVIS) tool to graphically present their evaluation of bias [21].

Synthesis of the Collected Data

The data analysis was conducted using Review Manager (RevMan) version 5.4 (The Cochrane Collaboration, Copenhagen, Denmark). The clinical and statistical heterogeneity of the included studies was assessed. To assess clinical heterogeneity, treatment protocols, inclusion criteria, participant characteristics, and outcome measures were compared. The chi-square test was used to evaluate statistical heterogeneity, with significance defined at p < 0.01. Heterogeneity between studies was also measured using the I² statistic [22]. For data analysis, mean differences (MDs) and their 95% confidence intervals were calculated using the inverse-variance method. Depending on the variability across studies, either a fixed-effects or a random-effects model was applied, and the results were presented as forest plots. When only one study addressed an outcome, or when outcomes were measured differently across studies, a qualitative synthesis was used instead. The overall certainty of the evidence was appraised using the GRADE framework. This methodology involves a structured evaluation of each outcome across key domains, including the volume of available studies, potential sources of bias, the consistency of observed results, the applicability of the evidence, and the precision of the estimated effects [23].

Results

Literature Review and Study Selection

**Figure 1:**
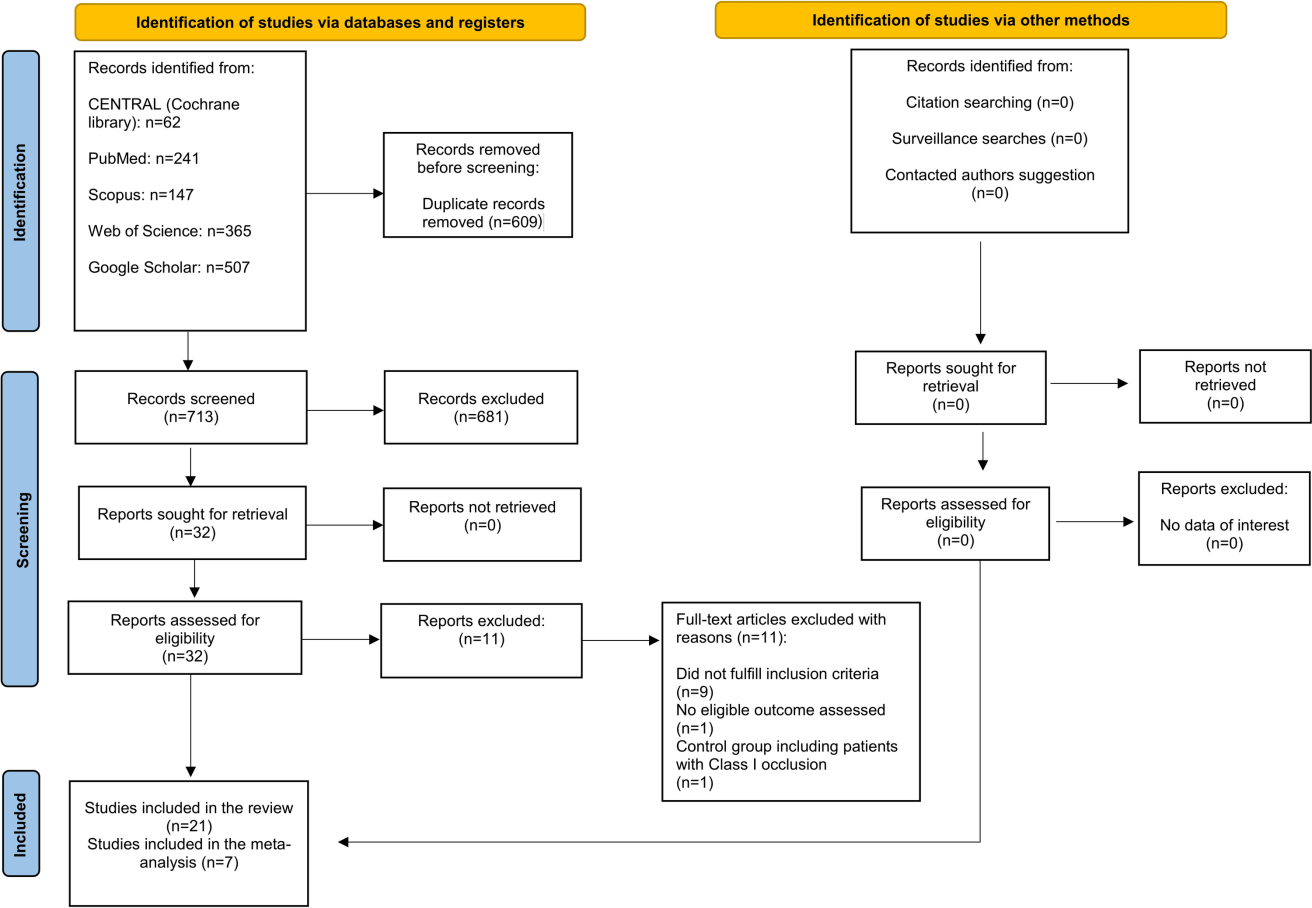
Preferred reporting items for systematic reviews and meta-analyses flow diagram of the included studies.

An electronic database search initially identified 1,322 studies. Following duplicate removal, 713 unique articles were retained for title and abstract screening against the predefined inclusion criteria, with ineligible studies excluded. Of these, 32 articles were selected for full-text review. After a detailed assessment, 11 studies were excluded because they did not meet the inclusion criteria. Ultimately, 21 studies [13,24-43] satisfied all eligibility criteria and were included in the systematic review. The study selection process is illustrated in the PRISMA flow diagram presented in Figure 1.

Characteristics of the Included Studies

Table 2 provides an overview of the features of the 21 studies included in this review. All of the studies, which were published between 1998 and 2025, were RCTs. The studies spanned a wide range of countries, including four papers from Syria, four from Turkey, three from China, two each from India and Egypt, and one trial each from the United Kingdom, Iran, Sweden, Germany, Brazil, and Malta. Out of the 21 reviewed studies, 18 focused on the skeletal type of anterior crossbite [13,24,25,27-30,32,33,35-43], whereas the functional and dental types were addressed in two [31,34] and one study [26], respectively. A total of 854 patients were included across the 21 studies. Among these, 30 patients presented with dental anterior crossbite, 86 with functional anterior crossbite, and 738 with skeletal anterior crossbite. The age of participants ranged from 6 to 12 years, with mean values between 7.9 and 11.5 years. Each study's sample size ranged from 24 to 73 patients, all of whom were growing children. All studies included both genders, except one that was confined to females [37].

**Table 2:** Characteristics of the included studies. M: male, F: female, CA: clear aligners, RIP: removable inclined plane, FM: face mask, MP: mentoplate, RME: rapid maxillary expansion, Alt-RAMEC: alternate rapid maxillary expansion and constriction, RTB: reversed twin block, PBMT: photobiomodulation therapy, LCP-IMT: lower-clear-plate based intermaxillary traction RTBLP: reverse twin block with lip pads, MTA: modified tandem appliance, RMR: removable mandibular retractor, BAIMT: bone-anchored intermaxillary traction, MSI: miniscrew implant, TTBA: tandem traction bow appliance, UCG: untreated control group, Lat. Ceph: lateral cephalometric, URA: Upper Removable Appliance, SNA: The angle between the anterior cranial base and NA plane, SNB: The angle between the anterior cranial base and NB plane, ANB: SNA minus SNB, Co: condylion, Gn: gnathion, Wits: distance between AO-BO, SN-GoMe: the angle between the anterior cranial base and the mandibular plane

Authors	Number of patients, M/F	Mean age	Intervention	Assessment method	Treatment duration	Assessed outcomes
Kiliçoglu and Kirliç (1998) [37]	Group 1: 16 f	8.65 ± 1.4 years	FM	Lat. Ceph	All treatment was finished once the anterior crossbite was corrected satisfactorily	SNA, SNB, ANB, SN-GoMe
	Group 2: 10 f	9.29 ± 1.4 years	UCG			
Xu and Lin (2001) [43]	Group 1: 20	9.3 years	FM-RME	Lat. Ceph	11.3 months	SNA, SNB, ANB, Co-Gn, SN-GoMe
	Group 2: 20		UCG			
Atalay and Tortop (2010) [27]	Group 1: 15 (6, 9)	8.18 ± 0.50 years	modified TTBA	Lat. Ceph	9 months	SNA, SNB, ANB, Co-Gn, overjet, overbite
	Group 2: 15 (10, 5)	7.90 ± 0.62 years	UCG		8 months	
	Group 3: 15 (10, 5)	11.75 ± 1.00 years	modified TTBA		11 months	
Abdelnaby and Nassar (2010) [24]	Group 1: 20 (10, 10)	9.6 years	600-g chin-cup and occlusal bite plane	Lat. Ceph	1 year	SNA, SNB, ANB, Wits appraisal, SN-GoMe
	Group 2: 20 (9, 11)	10.1 years	300-g chin-cup and occlusal bite plane			
	Group 3: 10 (5, 5)	9.2 years	UCG			
Mandall et al. (2010) [35]	Group 1: 35 (18, 17)	8.7 years	FM-RME	Lat. Ceph	6-12 months	SNA, SNB, ANB, overjet
	Group 2: 38 (16, 22)	9 years	UCG			
Showkatbakhsh et al. (2012) [33]	Group 1: 21 (10, 11)	8.9 ± 1.4 years	FM	Lat. Ceph	18 ± 2 months	SNA, SNB, ANB, Co-Gn
	Group 2: 21 (9, 12)	9.2 ± 1.1 years	Reverse chin cup		19 ± 4 months	
Ge et al. (2012) [28]	Group 1: 20 (9, 11)	10 years, 4 months	MSI + FM	Lat. Ceph	11 months	SNA, SNB, ANB, Wits appraisal, Co-Gn, SN-GoMe, overjet, overbite
	Group 2: 23 (11, 12)	10 years, 6 months	FM-RME		1 year, 1 month	
Wiedel and Bondemark (2015) [34]	Group 1: 31 (18, 13)	9.1 ± 1.19 years	URA with protruding springs	Study casts	6.9 ± 2.8 months	Overjet, overbite
	Group 2: 31 (19, 12)	10.4 ± 1.65 years	fixed appliance with multi-brackets		5.5 ± 1.41 months	
Canturk and Celikoglu (2015) [25]	Group 1: 15 patients (8, 7)	11.27 ± 1.26 years	Alt-RAMEC first, then FM	Lat. Ceph	7.33 ± 1.60	SNA, SNB, ANB, Wits appraisal, Co-Gn, overjet, overbite
	Group 2: 15 patients (6, 9)	10.53 ± 1.50 years	Alt-RAMEC + FM at the same time		5.76 ± 1.84	
Majanni and Hajeer (2016) [38]	Group 1: 19 (10, 9)	11.3 years	BAIMT	Lat. Ceph	12 months	SNA, SNB, ANB, Co-Gn, SN-GoMe, Overjet, Overbite
	Group 2: 19 (11, 8)	11.5 years	RMR			
Husson et al. (2016) [13]	Group 1: 16 (8, 8)	7.98 ± 0.68 years	MTA	Lat. Ceph	7.07 ± 0.78 months	SNA, SNB, ANB, Co-Gn, SN-GoMe, Overjet, Overbite
	Group 2: 16 (7, 9)	8.11 ± 0.76 years	FM + RME		6.4 ± 1.30 months	
Miamoto et al. (2018) [26]	Group 1: 15 (11, 4)	9.07 ± 0.79 years	URA with Finger Springs	Lat. Ceph + study casts	12 months	SNA, SNB, ANB, overjet
	Group 2: 15 (7, 8)	9.00 ± 0.84 years	Resin-reinforced GIC Bite Pads			
Minase et al. (2019) [32]	Group 1: 13 (3, 10)	10 ± 3.8 years	RTBLP-RME	Lat. Ceph	9 months	SNA, SNB, ANB, Wits appraisal, Co-Gn
	Group 2: 13 (6, 7)	10.2 ± 3.7 years	FM-RME			
	Group 3: 13 (6, 7)	10.3 ± 3.6 years	UCG			
James et al. (2020) [29]	Group 1: 26 (10, 16)	10.00 ± 1.24	AltRAMEC + Reverse Headgear	Lat. Ceph	6 months	SNA, SNB, ANB, overjet, overbite
	Group 2: 26 (14, 12)	10.23 ± 1.013	AltRAMEC + Reverse Headgear + Class III elastics			
Liang et al. (2021) [42]	Group 1: 20 (8, 12)	10.75 ± 1.3 years	FM anchored with customized miniplates	CBCT	10.6 months	SNA, SNB, ANB, SN-GoMe
	Group 2: 21 (11, 10)	10.5 ± 1.1 years	FM anchored with teeth		12.1 months	
Alzabibi et al. (2021) [40]	Group 1: 21 (12, 9)	8.95 ± 0.88 years	LCP-IMT + RME	Lat. Ceph	4.34 ± 2.02 months	SNA, SNB, ANB, Wits appraisal, Co-Gn, SN-GoMe, Overjet, Overbite
	Group 2: 19 (8, 11)	9.14 ± 0.80 years	UCG		6 months	
Khwanda et al. (2022) [41]	Group 1: 20 (12, 8)	10.27± 0.80 years	RTB + PBMT	CBCT images	6.4 months	SNA, SNB, ANB
	Group 2: 20 (14, 6)	10.12 ± 0.84 years	RTB		8.6 months	
Alzoubi et al. (2023) [30]	Group 1: 17 (12, 5)	8.2 ± 0.6 years	Tooth-borne FM + Alt-RAMEC	Lat. Ceph	9 months	SNA, SNB, ANB, Wits appraisal, Co-Gn, Overjet, Overbite
	Group 2: 17 (11, 6)	8.8 ± 0.8 years	Skeletally anchored FM + Alt-RAMEC			
Yavan et al. (2023) [39]	Group 1: 15 (7, 8)	10.54 years	FM-RME	Lat. Ceph	9.25 ± 0.91 months	SNA, SNB, ANB, Overjet, Overbite
	Group 2: 15 (9, 6)	10.49 years	Reverse Forsus		4.54 ± 0.93 months	
	Group 3: 15 (8, 7)	10.66 years	UCG		6.10 ± 0.15 months	
Meyns et al. (2025) [36]	Group 1: 14 (7, 7)	9.6 years	Facemask + Hybrid Hyrax	Low-dose computed tomography (CT) scans	12 months	SNA, SNB, ANB, Wits appraisal, SN-GoMe
	Group 2: 14 (7, 7)	9.7 years	Mentoplate + Hybrid Hyrax		11.7 months	
Salem et al. (2025) [31]	Group 1: 12 (8,4)	10.74 ± 1.1 years	CA	Digital models	4 months	Overjet, Overbite
	Group 2: 12 (8,4)	10.54 ± 1.06 years	RIP			

Nine studies evaluated the effectiveness of intraoral non-skeletally anchored appliances for managing anterior crossbite. Six of these focused on correcting skeletal mandibular anterior crossbite. Among them, one study assessed the Reverse Forsus appliance compared with facemask therapy combined with rapid maxillary expansion (FM-RME) and an untreated control group [39]. Two studies examined the reverse twin block (RTB): one compared RTB with facemask therapy and untreated controls [32], while the other investigated RTB combined with photobiomodulation therapy versus RTB alone [41]. Another study evaluated the lower clear-plate intermaxillary traction (LCP-IMT) appliance against an untreated control group [40]. Two additional studies analyzed the modified tandem appliance: one compared it with FM-RME [13], and the other assessed outcomes across two age groups relative to untreated controls [27].

Three further studies addressed the correction of dental and functional anterior crossbite. Salem et al. compared clear aligners with a removable inclined plane [31]; Miamoto et al. compared resin-reinforced glass ionomer cement bite pads with an upper removable appliance featuring finger springs [26]; and Wiedel and Bondemark compared a removable appliance with protruding springs to a fixed multibracket system [34].

Regarding extraoral non-skeletally anchored appliances, seven studies were identified. Two studies evaluated FM-RME compared with untreated controls [35,43], while two others examined facemask therapy without expansion, one against reverse chincup therapy [33] and the other against an untreated group [37]. Two additional trials compared immediate versus delayed traction using the alternate rapid maxillary expansion and constriction (Alt-RAMEC) strategy in conjunction with facemask treatment [25] and assessed the addition of Class III elastics [29]. The final study investigated chincup therapy at two force levels relative to untreated controls [24].

Three studies evaluated extraoral skeletally anchored appliances. Liang et al. compared a miniplate-supported facemask with a conventional tooth-borne facemask [42], while Ge et al. examined a miniscrew-anchored facemask versus a traditional facemask with RME [28]. Alzoubi et al. investigated a skeletally anchored Alt-RAMEC protocol compared with a tooth-borne approach [30]. Two studies assessed intraoral skeletally anchored appliances, including a mentoplate with a hybrid hyrax expander compared with a facemask employing the same expander [36] and bone-anchored intermaxillary traction (BAIMT) versus the RMR [38].

Across the included studies, diverse assessment methods were employed; most commonly, lateral cephalometric images were used in 16 studies [13,24,25,27-30,32,33,35,37-40,43]. Cone-beam computed tomography (CBCT), one of the more sophisticated imaging methods, was used in two studies [41,42], and low-dose computed tomography (CT) was used in one study [36] to evaluate changes in SNA°, SNB°, ANB°, Wits appraisal, overbite, and overjet. Additionally, three studies employed study casts or digital dental models to assess changes in overjet and overbite [26,31,34]; one of these studies incorporated study casts alongside lateral cephalometric images [26].

Risk of Bias of the Involved Studies

**Figure 2:**
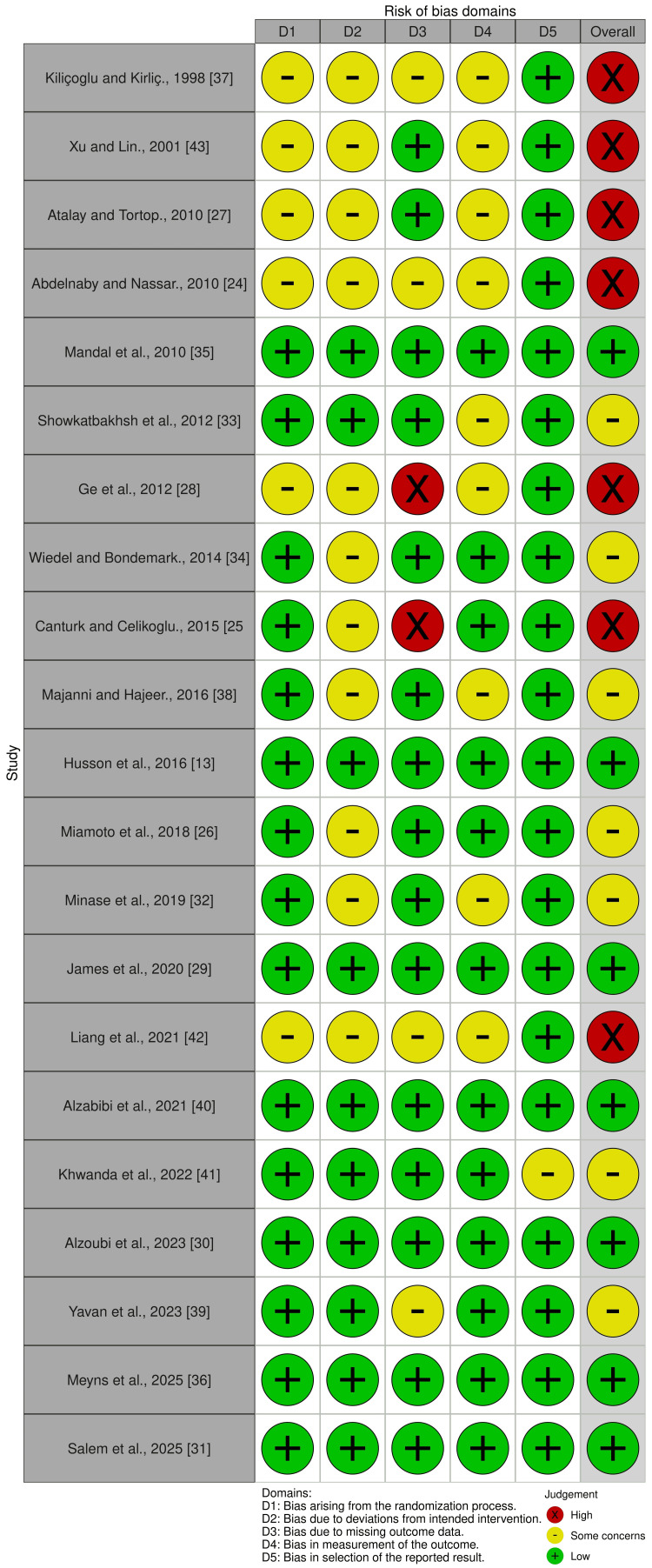
Risk-of-bias summary: review authors' judgments for each risk-of-bias domain across the included studies.

**Figure 3:**
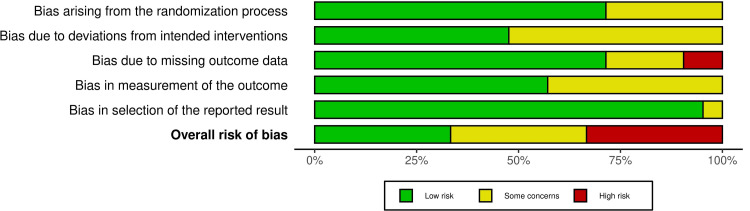
Risk of bias graph: the judgments of the review authors regarding each risk of bias domain, displayed as percentages across all the studies involved.

The risk-of-bias evaluations for the included RCTs are shown in Figure 2, and the overall risk of bias across all domains is summarized in Figure 3. Comprehensive evaluation criteria and justifications for each judgment are provided in Table 3. Of the studies reviewed, seven RCTs were assessed as having a low risk of bias [13,30,31,35,36,40]. An additional seven studies were deemed to present some concerns regarding bias [26,32-34,38,39,41]. The remaining seven studies were classified as being at high risk of bias [24,25,27,28,37,42,43]. The reasons for the high risk of bias in these seven RCTs included deficiencies in the randomization process [24,27,28,37,42,43], deviations from the intended interventions [24,25,27,28,37,42,43], missing outcome data [24,25,28,37,42], and failure to blind the outcome assessor [24,27,28,37,42,43].

**Table 3:** Details of the risk of bias assessment of the studies using the Cochrane's Risk of Bias tool 2.0.

Study	Bias arising from the randomization process	Bias due to deviations from intended interventions	Bias due to missing outcome data	Bias in the measurement of the outcome	Bias in the selection of the reported result	Overall bias
	D1	D2	D3	D4	D5	
Salem et al. (2025) [31]	Low risk: “A box containing 24 cards, each card had the name of one of the two treatment protocols used. A card was drawn by each participant at random after shaking the box; the participant was then allocated according to the indicated group.”	Low risk: Complete blinding was not possible since the operator and patients were aware of the specific type of appliance utilized in each case. We judge that the outcome is not likely to be influenced by the lack of blinding.	Low risk: No dropouts were reported.	Low risk: “The outcome assessor remained blinded, and the data were sent without identifiers for Statistical analysis.”	Low risk: This study was recorded in the ClinicalTrials.gov database (identifier: NCT06015386), and the outcomes mentioned in the protocol have been reported.	Low risk
Meyns et al. (2025) [36]	Low risk: “a. Sequence generation: The randomization sequence was generated with a 1:1 allocation ratio. b. Allocation concealment: Sequentially numbered sealed, opaque envelopes. c. Implementation: The envelopes containing the allocation sequence codes were given to the patient by an intermediary and opened sequentially at the time of enrolment, excluding the clinician from the process.”	Low risk: “Due to the nature of the trial, the operator and children could not be blinded to the treatment allocation.” We judge that the outcome is not likely to be influenced by the lack of blinding.	Low risk: “One patient, initially assigned to the MP group, was later excluded due to non-cooperation, leading to discontinuation of treatment.” Nearly all the outcome data is available (97%).	Low risk: The outcome assessor was blinded	Low risk: “This Randomized Clinical trial was registered at www.ClinicalTrials.gov (ID: NCT02711111)”, and the outcomes mentioned in the protocol have been reported.	Low risk
Yavan et al. (2023) [39]	Low risk: “The 45 participants were randomly divided into three groups based on age and gender by a statistician using computer software. Following randomization, baseline balance testing was performed. Concealed allocation was performed using opaque, sealed envelopes containing the patient list of each group.”	Low risk: “It was not possible to blind the patients and the researcher who administered the treatment. We judge that the outcome is not likely to be influenced by the lack of blinding.”	Some concerns: About 11% of the participants were lost to follow-up. 6 of 51 participants. The reason for the exclusion is somewhat unconvincing.	Low risk: “The researcher who performed the cephalometric measurements and the statistician who conducted the statistical analyses after treatment were blinded to the clinical data of the groups.“	Low risk: No information about the registration protocol was mentioned. However, the predefined outcomes mentioned in the methods section seemed to be reported.	Some concerns
Alzoubi et al. (2023) [30]	Low risk: “A randomization tool (Randomizer software) was used for assignation. The software generated codes for each patient to pseudonymise the study and randomly allocate the patients 1:1 into one of two groups. The sealed envelope technique was used to ensure randomization. The allocation sequence codes were contained within opaque envelopes.”	Low risk: “Because of the character of the trial, the operator and children could not be blinded to treatment allocation.” We judge that the outcome is not likely to be influenced by the lack of blinding.	Low risk: “One patient in group I developed leukemia early in treatment and was unable to continue.” Nearly all the outcome data is available (97%).	Low risk: The outcome assessor was blinded	Low risk: “The study was registered as a Randomized Clinical Trial (RCT), https://doi.org/10.1186/ISRCTN12197405.” The outcomes mentioned in the protocol have been reported.	Low risk
Khwanda et al. (2022) [41]	Low risk: “Patients were assigned to the irradiation group (RTB+PBMT) or the control group (RTB) with an allocation ratio of 1:1 using a simple randomization technique. Each patient was asked to select a folded piece of paper from a box containing 40 pieces of paper.”	Low risk: “Blinding of patients or the principal researcher was impossible.” We judge that the outcome is not likely to be influenced by the lack of blinding.	Low risk: No dropouts were reported.	Low risk: The outcome assessor was blinded.	Some concerns: The protocol for the study was registered on the German Clinical Trials Register (DRKS-ID: DRKS00027535), but the outcomes mentioned in the protocol have not all been reported.	Some concerns
Alzabibi et al. (2021) [40]	Low risk: “The randomization sequence was computer-generated. The allocation sequence was concealed using sequentially opaque, sealed envelopes.”	Low risk: “Due to the nature of the trial, blinding of the patients and the clinicians was not applicable.” We judge that the outcome is not likely to be influenced by the lack of blinding.	Low risk: Two patients were lost to follow-up due to personal reasons. Nearly all the outcome data is available (95%).	Low risk: The outcome assessor was blinded.	Low risk: The protocol for the study was registered in clinicaltrials.gov study ID: NCT 03172442, and the outcomes mentioned in the protocol have been reported.	Low risk
Liang et al. (2021) [42]	Some concerns: No details about randomization.	Some concerns: No details about blinding, either for the patient or the clinician	Some concerns: No details of data dropouts	Some concerns: No details of the blinding of outcome assessors were reported.	Low risk: No information about the registration protocol was mentioned. However, the predefined outcomes mentioned in the methods section seemed to be reported.	High risk
James et al. (2020) [29]	Low risk: “Subjects were randomly allotted to group 1 or group 2 with an allocation ratio of 1:1. For the execution of a planned single-blinded study, blocks of random numbers were assigned using computer-generated tables.”	Low risk: “Blinding was possible only with regard to evaluation of cephalometric radiographs.” We judge that the outcome is not likely to be influenced by the lack of blinding.	Low risk: No dropouts were reported.	Low risk: “The tracings were performed randomly, by a blinded examiner."	Low risk: No information about the registration protocol was mentioned. However, the predefined outcomes mentioned in the methods section seemed to be reported.	Low risk
Minase et al. (2019) [32]	Low risk: “Simple randomization was performed by creating a randomization list using Minitab® V16 with an allocation ratio of 1:1:1.”	Some concerns: No details about blinding, either for the patient or the clinician.	Low risk: No dropouts were reported.	Some concerns: No details of the blinding of outcome assessors were reported.	Low risk: No information about the registration protocol was mentioned. However, the predefined outcomes mentioned in the methods section seemed to be reported.	Some concerns
Miamoto et al. (2018) [26]	Low risk: “The distribution of the 30 individuals into the two groups was performed in a randomized manner as follows: a sealed envelope was prepared with 30 cards containing the names of the two treatment protocols on 15 cards each.”	Some concerns: No details about blinding, either the patient or the clinician.	Low risk: No dropouts were reported.	Low risk: The outcome assessor was blinded.	Low risk: No information about the registration protocol was mentioned. However, the predefined outcomes mentioned in the methods section seemed to be reported.	Some concerns
Husson et al. (2016) [13]	Low risk: “Patients were randomized in a 1:1 ratio into two equal groups using an online randomization service.”	Low risk: “Blinding of either patient or operator was not possible.” We judge that the outcome is not likely to be influenced by the lack of blinding.	Low risk: No dropouts were reported.	Low risk: The outcome assessor was blinded.	Low risk: The protocol for the study was registered in clinicaltrials.gov study ID: NCT02144324, and the outcomes mentioned in the protocol have been reported.	Low risk
Majanni and Hajeer (2016) [38]	Low risk: “The randomization sequence was computer-generated. The allocation sequence was concealed using sequentially opaque, sealed envelopes.”	Some concerns: No details about blinding, either the patient or the clinician.	Low risk: No dropouts were reported.	Some concerns: No details of the blinding of outcome assessors were reported.	Low risk: No information about the registration protocol was mentioned. However, the pre-defined outcomes mentioned in the methods section seemed to be reported.	Some concerns
Canturk and Celikoglu (2015) [25]	Low risk: “Thirty-six patients who met the above criteria were divided into two groups using a randomization method with pitch and toss.”	Some concerns: No details about blinding, either the patient or the clinician.	High risk: Six patients were lost to follow-up due to a lack of cooperation. About 17% of the participants were lost	Low risk: The outcome assessor was blinded.	Low risk: No information about the registration protocol was mentioned. However, the predefined outcomes mentioned in the methods section seemed to be reported.	High risk
Wiedel and Bondemark (2015) [34]	Low risk: “The subjects were randomized by an independent person in blocks of 10, as follows: seven opaque envelopes were prepared with 10 sealed notes in each (5 notes for each group).”	Some concerns: No details about blinding, either the patient or the clinician.	Low risk: “62 patients were randomized into the two groups and all but one completed the trial.” Nearly all the outcome data is available (99%).	Low risk: The outcome assessor was blinded.	Low risk: No information about the registration protocol was mentioned. However, the predefined outcomes mentioned in the methods section seemed to be reported.	Some concerns
Ge et al. (2012) [28]	Some concerns: No details about randomization.	Some concerns: No details about blinding, either the patient or the clinician.	High risk: “During treatment, four subjects of the initial MSI/FM group were excluded because of the mobility of miniscrews, and one subject of each group was excluded because of poor cooperation.” About 20% of the participants were lost	Some concerns: No details of the blinding of outcome assessors were reported.	Low risk: No information about the registration protocol was mentioned. However, the predefined outcomes mentioned in the methods section seemed to be reported.	High risk
Showkatbakhsh et al. (2012) [33]	Low risk: “An unstratified subject allocation sequence was generated by a computer program (Etcetra Version 2.59); random numbers were generated.”	Low risk: “The treating clinician was blinded from the randomization procedure, but because of clear differences in appliance design, blinding was not possible during the treatment period.” We judge that the outcome is not likely to be influenced by the lack of blinding.	Low risk: No dropouts were reported.	Some concerns: No details of the blinding of outcome assessors were reported.	Low risk: No information about the registration protocol was mentioned. However, the predefined outcomes mentioned in the methods section seemed to be reported.	Some concerns
Mandall et al. (2010) [35]	Low risk: “The randomization list was generated in randomization blocks of 10 with stratification according to gender. The computer-generated randomization sequence was concealed centrally.”	Low risk: “It was not possible to blind the clinician or the patient in this study.” We judge that the outcome is not likely to be influenced by the lack of blinding.	Low risk: Four patients were lost to follow-up. Nearly all the outcome data is available (95%).	Low risk: The outcome assessor was blinded.	Low risk: No information about the registration protocol was mentioned. However, the predefined outcomes mentioned in the methods section seemed to be reported.	Low risk
Abdelnaby and Nassar (2010) [24]	Some concerns: No details about randomization.	Some concerns: No details about blinding, either the patient or the clinician.	Some concerns: No details of data dropouts.	Some concerns: No details of the blinding of outcome assessors were reported.	Low risk: No information about the registration protocol was mentioned. However, the predefined outcomes mentioned in the methods section seemed to be reported.	High risk
Atalay and Tortop (2010) [27]	Some concerns: No details about randomization.	Some concerns: No details about blinding, either the patient or the clinician.	Low risk: No dropouts were reported.	Some concerns: No details of the blinding of outcome assessors were reported.	Low risk: No information about the registration protocol was mentioned. However, the predefined outcomes mentioned in the methods section seemed to be reported.	High risk
Xu and Lin (2001) [43]	Some concerns: No details about randomization.	Some concerns: No details about blinding, either the patient or the clinician.	Low risk: No dropouts were reported.	Some concerns: No details of the blinding of outcome assessors were reported.	Low risk: No information about the registration protocol was mentioned. However, the predefined outcomes mentioned in the methods section seemed to be reported.	High risk
Kiliçoglu and Kirliç (1998) [37]	Some concerns: No details about randomization.	Some concerns: No details about blinding, either the patient or the clinician.	Some concerns: No details of data dropouts.	Some concerns: No details of the blinding of outcome assessors were reported.	Low risk: No information about the registration protocol was mentioned. However, the predefined outcomes mentioned in the methods section seemed to be reported.	High risk

Effects of Intervention

The primary findings of the selected studies included in the review are presented in Table 4.

**Table 4:** Main findings of the included studies. CA: clear aligners, RIP: removable inclined plane, FM: face mask, MP: mentoplate, RME: rapid maxillary expansion, Alt-RAMEC: alternate rapid maxillary expansion and constriction, RTB: reversed twin block, PBMT: photobiomodulation therapy, LCP-IMT: lower-clear-plate-based intermaxillary traction, RTBLP: reverse twin block with lip pads, MTA: modified tandem appliance, RMR: removable mandibular retractor, BAIMT: bone-anchored intermaxillary traction, MSI: miniscrew implant, TTBA: tandem traction bow appliance, UCG: untreated control group, Lat. Ceph: lateral cephalometric, URA: Upper Removable Appliance, SNA: The angle between the anterior cranial base and NA plane, SNB: The angle between the anterior cranial base and NB plane, ANB: SNA minus SNB, Wits: distance between AO-BO, Co-Gn: the length of the mandible, SN-GoMe: the angle between the anterior cranial base and the mandibular plane

Authors (year)	Groups	Mean differences following treatment							
		SNA	SNB	ANB	Wits appraisal	Co-Gn	SN-GoMe	Overjet	Overbite
Kiliçoglu and Kirliç (1998) [37]	FM UCG	2.56°, 0.05°	-1.78°, 0.44°	4.34°, -0.28°	-	-	1.75°, -0.61°	-	-
Xu and Lin (2001) [43]	FM-RME UCG	1.25°, -0.19°	-1.69°, 1.25°	3°, -1.5°	-	1 mm, 1.94 mm	2.31°, -1.13°	-	-
Atalay and Tortop (2010) [27]	Modified TTBA UCG	0.7°, 0.5°	-1.1°, 0.4°	1.7°, 0°	-	1.9 mm, 2.9 mm	-	3.6 mm, 0.3 mm	-1.2 mm, 0.3 mm
Abdelnaby and Nassar (2010) [24]	Chin cup using 600 g, chin cup using 300g UCG	0.3°, 0.4° 0.2°	-2.20°, -2° -0.3°	2.5°, 2.4°, 0.5°	4.6 mm, 4.9 mm, -0.20 mm	-	1.5°, 1.4°, 0.5°	-	-
Mandal et al. (2010) [35]	FM-RME UCG	1.4°, 0.3°	-0.7°, 0.8°	2.1°, -0.5°	-	-	-	4.4 mm, 0.3 mm	-
Showkatbakhsh et al. (2012) [33]	FM Reverse chin cup	1°, 1.8°	-0.5°, 0.3°	1.6°, 1.4°	-	1.5 mm, 1.6 mm	-	-	-
Ge et al. (2012) [28]	MSI + FM FM-RME	2.58°, 2.62°	1.80°, 1.79°	4.37°, 4.42°	4.83 mm, 5.33 mm	2.81 mm, 3.84 mm	1.84°, 1.98°	6.19 mm, 5.79 mm	-2.03 mm, -2.90 mm
Wiedel and Bondemark (2014) [34]	URA with protruding springs, fixed appliance with multi-brackets	-	-	-	-	-	-	3.5 mm, 4.2 mm	-0.1 mm, 0 mm
Canturk and Celikoglu (2015) [25]	Alt-RAMEC first, then FM Alt-RAMEC + FM at the same time	3.70°, 3.68°	-1.91°, -1.54°	5.65°, 5.25°	5.37 mm, 4.86 mm	0.85 mm, 0.70 mm	-	6.92 mm, 6.70 mm	-1.96 mm, -2.58 mm
Majanni and Hajeer (2016) [38]	BAIMT RMR	1.20°, 0.48°	-1.56°, -0.76°	2.76°, 1.24°	-	2.62 mm, 2.86 mm	1.05°, -0.08°	4.57 mm, 3.95 mm	-1.60 mm, -1.72 mm
Husson et al. (2016) [13]	MTA FM + RME	1.38°, 1.5°	-0.44°, -0.69°	1.88°, 2.13°	-	1.13 mm, 1.25 mm	1.00°, 3.5°	2.25 mm, 3.53 mm	-0.4 mm, -1.76 mm
Miamoto et al. (2018) [26]	URA with Finger Springs Resin-reinforced GIC Bite Pads	1.06°, -0.14°	0.78°, 0°	0.29°, -0.14°	-	-	-	1.40 mm, 1.00 mm	-
Minase et al. (2019) [32]	RTBLP-RME FM-RME UCG	2°, 1.31°, 0.81°	-1.08°, -0.73°, 1.38°	3.08°, 2.04°, -0.58°	4.23 mm, 2.38 mm, -1.04 mm	4.38 mm, 5.85 mm, 3.62 mm	-	-	-
James et al. (2020) [29]	AltRAMEC + Reverse Headgear AltRAMEC + Reverse Headgear + Class III elastics	2.93°, 5.45°	-0.38°, -1.07°	3.31°, 4.66°	-	-	-	4.88 mm, 8.22 mm	-0.23 mm, -2.41 mm
Liang et al. (2021) [42]	FM anchored with customized miniplates, FM anchored with teeth	2.65°, 1.85°	-0.49°, -0.84°	3.14°, 2.69°	-	-	0.09°, 1.08°	-	-
Alzabibi et al. (2021) [40]	LCP-IMT + RME UCG	1.31°, 0.29°	-1.85°, 0.97°	3.12°, -0.69°	5.24 mm, -0.72 mm	0.58 mm, 1.61 mm	1.12°, -0.85°	5.87 mm, -0.33 mm	0.62 mm, 0.21 mm
Khwanda et al. (2022) [41]	RTB + PBMT RTB	1.93°, 1.54°	-1°, -0.88°	2.9°, 2.43°	-	-	-	-	-
Alzoubi et al. (2023) [30]	Tooth-borne FM + Alt-RAMEC, Skeletally anchored FM + Alt-RAMEC	2.10°, 2.50°	-1.40°, 0.00°	3.90°, 3.10°	4.70 mm, 3.20 mm	3.70 mm, 3.00 mm	-	5.40 mm, 4.50 mm	1.90 mm, 1.00 mm
Yavan et al. (2023) [39]	FM-RME Reverse Forsus UCG	2.82°, 1.32° 0.40°	-1.12°, -1.08° 0.67°	3.95°, 2.36° -0.48°	-	-	-	5.91 mm, 5.45 mm, -0.09 mm	-0.93 mm, -1.96 mm, 0.24 mm
Meyns et al. (2025) [36]	Facemask + Hybrid Hyrax, Mentoplate + Hybrid Hyrax	2.48°, 1.99°	-0.86°, -0.64°	3.36, 2.63°	4.42 mm, 2.86 mm	-	0.52°, -0.46°	-	-
Salem et al. (2025) [31]	CA RIP	-	-	-	-	-	-	2.92 mm, 3.44 mm	4.24 mm, 4.57 mm

Intraoral non-skeletally anchored appliances: Across the included studies, intraoral non-skeletally anchored appliances generally produced modest improvements in sagittal jaw relationships, with ANB angle changes ranging from approximately 0.29° to 3.1° [13,26,27,32,39,41]. The greatest increase was reported by Alzabibi et al. (MD: 3.12°) [40], while the remaining appliances - including the RTB, Reverse Forsus, and MTA devices - demonstrated increases within a similar range. Regarding maxillary position (SNA angle), Minase et al. reported the greatest mean increase (2°) with the RTB device [32], followed by Khwanda et al. (1.54°) [41]. The remaining five studies showed mean increases ranging from 0.7° to 1.38° [13,26,27,39,40].

For mandibular position (SNB angle), Alzabibi et al. reported the most pronounced mean decrease (1.85°) with the LCP-IMT [40]. Conversely, Miamoto et al. observed a mean increase of 0.78° with a removable appliance with a finger spring [26]. The remaining five studies reported mean reductions ranging from 0.44° to 1.08° [13,27,32,39,41].

Regarding overjet changes, intraoral appliances produced a broad range of improvements, typically between about 1.4 mm and 5.9 mm [13,26,27,31,34,39]. The largest increase was reported by Alzabibi et al. (MD: 5.87 mm) [40]. 

**Figure 4:**
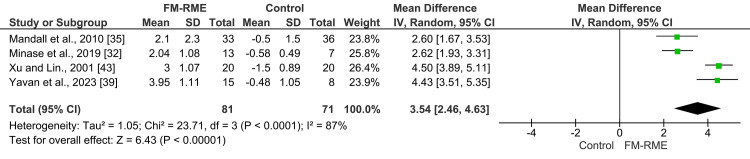
A forest plot illustrating changes in the ANB angle produced by face mask therapy combined with rapid maxillary expansion compared with the control group.

Extra-oral non-skeletally anchored appliances: Nine studies examined the effects of extra-oral non-skeletally anchored appliances on the sagittal relationship between the two jaws (ANB angle). Due to considerable variability in the devices employed, only four of the nine studies were incorporated into the meta-analysis [32,35,39,43]. The pooled estimate of four studies (n = 152) using the random-effects model showed that using the FM-RME resulted in a significant mean increase in the ANB angle of 3.54° (MD = 3.54°; 95% CI: 2.46, 4.63; p < 0.00001; χ² = 23.71; p ˂ 0.0001; I² = 87%; Figure 4) [32,35,39,43]. Based on the GRADE assessment, the quality of evidence for this outcome was considered low (Table 5). A leave-one-out sensitivity analysis was carried out to determine how each individual study affected the overall results; however, substantial heterogeneity persisted. Despite this variability, all studies consistently demonstrated a beneficial effect of the facemask, justifying the presentation of the pooled results.

**Table 5:** Summary of findings table according to the Grading of Recommendations, Assessment, Development, and Evaluation guidelines for the included trials. High quality: Further research is very unlikely to change our confidence in the estimate of effect; Moderate quality: Further research is likely to have an important impact on our confidence in the estimate of effect and may change the estimate; Low quality: Further research is very likely to have an important impact on our confidence in the estimate of effect and is likely to change the estimate; Very low quality: we are very uncertain about the estimate. ^a^ Decline one level for risk of bias, and one level for Inconsistency; ^b^ Decline one level for risk of bias; ^c^ Decline one level for risk of bias, and one level for Inconsistency; ^d^ Decline one level for risk of bias; ^e^ Decline one level for risk of bias and one level for Inconsistency; ^f^ Decline one level for risk of bias; ^g^ Decline one level for risk of bias, one level for Inconsistency, and one level for Imprecision. CI: confidence interval, GRADE: Grading of Recommendations, Assessment, Development, and Evaluation, RCT: randomized controlled clinical trial, SNA: The angle between the anterior cranial base and NA plane, SNB: The angle between the anterior cranial base and NB plane, ANB: SNA minus SNB

Quality assessment criteria							Summary of findings				Comments
Comparison	No. of studies	Risk of bias	Inconsistency	Indirectness	Imprecision	Other considerations	No. of patients	Effects		Certainty	
								Absolute (95% CI)	Relative (95% CI)		
ANB change: FM-RME VS Control	4 RCTs	Serious	Serious	Not serious	Not serious	None	152	-	Mean 3.54° CI 95% (2.46, 4.63)	⊕⊕OO ^a^ Low	The difference was significantly higher on the intervention side than on the control side, with a low quality of evidence ⊕⊕OO ^a^
ANB change: Skeletally anchored FM VS Tooth-anchored FM	3 RCTs	Serious	Not serious	Not serious	Not serious	None	118	-	Mean 0.07° CI 95% (-0.57, 0.72)	⊕⊕⊕O ^b^ Moderate	There was no significant difference between the two groups, with a moderate quality of evidence ⊕⊕⊕O ^b^
SNA change: FM-RME VS Control	4 RCTs	Serious	Serious	Not serious	Not serious	None	152	-	Mean 1.37° CI 95% (0.57, 2.16)	⊕⊕OO ^c^ Low	The difference was significantly higher on the intervention side than on the control side, with a low quality of evidence ⊕⊕OO ^c^
SNA change: Skeletally anchored FM VS Tooth-anchored FM	3 RCTs	Serious	Not serious	Not serious	Not serious	None	118	-	Mean 0.59° CI 95% (0.02, 1.16)	⊕⊕⊕O ^d^ Moderate	The difference was significantly higher on the skeletally anchored FM side than on the Tooth-anchored FM side, with a moderate quality of evidence ⊕⊕⊕O ^d^
SNB change: FM-RME VS Control	4 RCTs	Serious	Serious	Not serious	Not serious	None	152	-	Mean -2.14° CI 95% (-2.93, -1.35)	⊕⊕OO ^e^ Low	The differences were significantly higher on the intervention side than on the control sides with a low quality of evidence ⊕⊕OO ^c^
SNB change: Skeletally anchored FM VS Tooth-anchored FM	3 RCTs	Serious	Not serious	Not serious	Not serious	None	118	-	Mean 0.24° CI 95% (-0.31, 0.79)	⊕⊕⊕O ^f^ Moderate	There was no significant difference between the two groups, with a moderate quality of evidence ⊕⊕⊕O ^f^
Overjet change: Skeletally anchored FM VS Tooth-anchored FM	2 RCTs	Serious	Serious	Not serious	Serious	None	77	-	Mean -0.19° CI 95% (-1.46 1.08)	⊕OOO ^g^ Very low	There was no significant difference between the two groups, with a very low quality of evidence ⊕OOO ^g^

**Figure 5:**
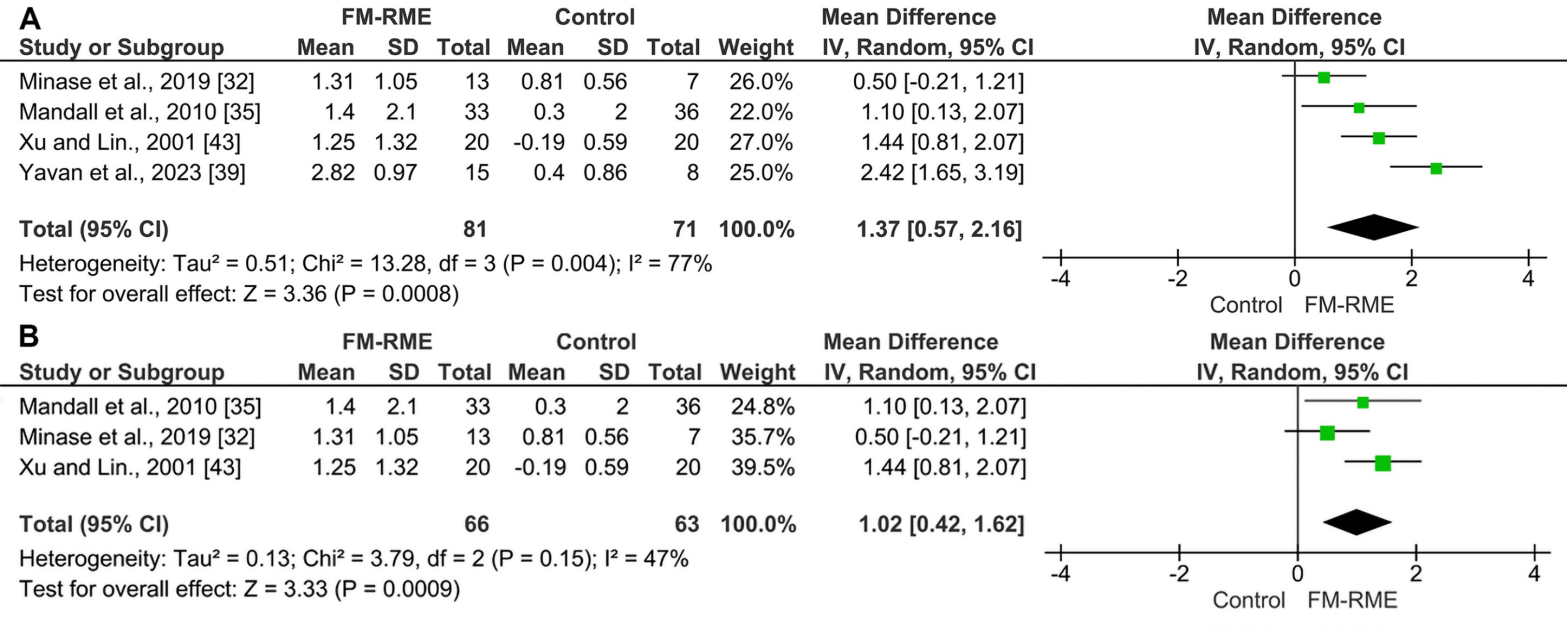
A forest plot illustrating the changes in the SNA angle produced by face mask therapy combined with rapid maxillary expansion compared with the control group. A) The original test; B) After performing a leave-one-out sensitivity test.

For maxillary position (SNA angle), the pooled estimate of four studies (n = 152) using the random-effects model showed that using the FM-RME resulted in a significant mean increase of 1.37° in the SNA angle (MD = 1.37°; 95% CI: 0.57, 2.16; p = 0.0008; χ² = 13.28; p = 0.004; I² = 77%; Figure 5A) [32,35,39,43]. According to the GRADE approach, the evidence quality for this outcome was rated as low. A leave-one-out sensitivity analysis was performed to examine the effect of individual studies on the pooled results, revealing that the study by Yavan et al. [39] had the greatest impact on heterogeneity. After excluding this study, the revised meta-analysis still demonstrated that FM-RME use was associated with a significant mean increase of 1.02° in the SNA angle, while heterogeneity was reduced to 47% (MD = 1.02°; 95% CI: 0.42, 1.62; p = 0.0009; χ² = 3.79; p = 0.15; I² = 47%; Figure 5B).

**Figure 6:**
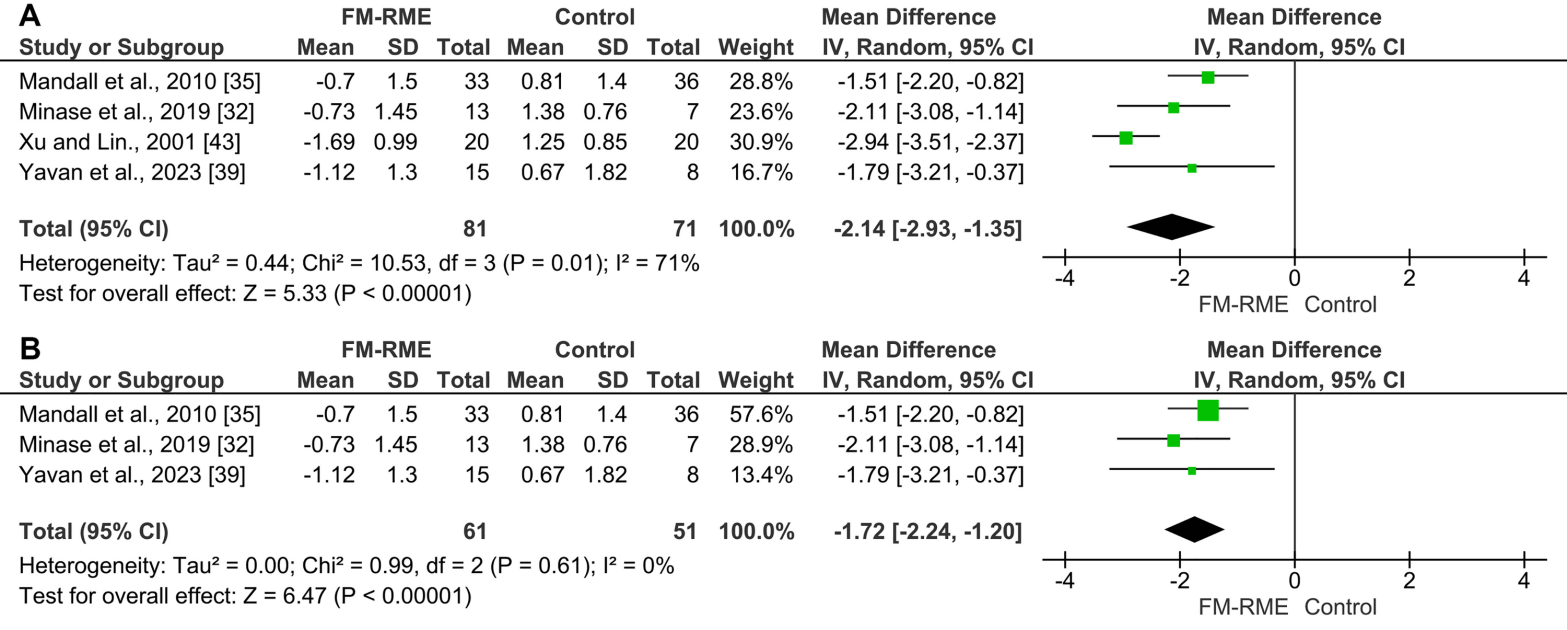
A forest plot illustrating the changes in the SNB angle produced by face mask therapy combined with rapid maxillary expansion compared with the control group. A) The original test; B) After performing a leave-one-out sensitivity test.

Regarding mandibular position (SNB angle), the pooled estimate of four studies (n = 152) using the random-effects model showed that using the FM-RME resulted in a significant mean decrease in the SNB angle of 2.14° (MD = -2.14°; 95% CI: -2.93, -1.35; p < 0.00001; χ² = 10.53; p = 0.01; I² = 71%; Figure 6A) [32,35,39,43]. According to GRADE, the quality of evidence for this outcome was low. A leave-one-out sensitivity analysis was performed. The analysis identified Xu and Lin's study as exerting the greatest influence on heterogeneity [43]. Upon exclusion of this study, the recalculated meta-analysis continued to demonstrate a significant mean reduction of 1.72° in the SNB angle associated with the use of FM-RME, and the heterogeneity was reduced to 0% (MD = -1.72°; 95% CI: -2.24, -1.20; p < 0.00001; χ² = 0.99; p = 0.61; I² = 0%; Figure 6B).

Three studies assessed overjet changes. James et al. reported a significantly greater mean increase in the Class III elastic group (8.22 mm) compared to the traditional FM group (4.88 mm) [29]. Canturk and Celikoglu found no significant difference between simultaneous and delayed FM application (6.70 mm vs. 6.92 mm) [25]. Mandal et al. reported a mean increase of 4.4 mm following FM-RME therapy [35].

**Figure 7:**
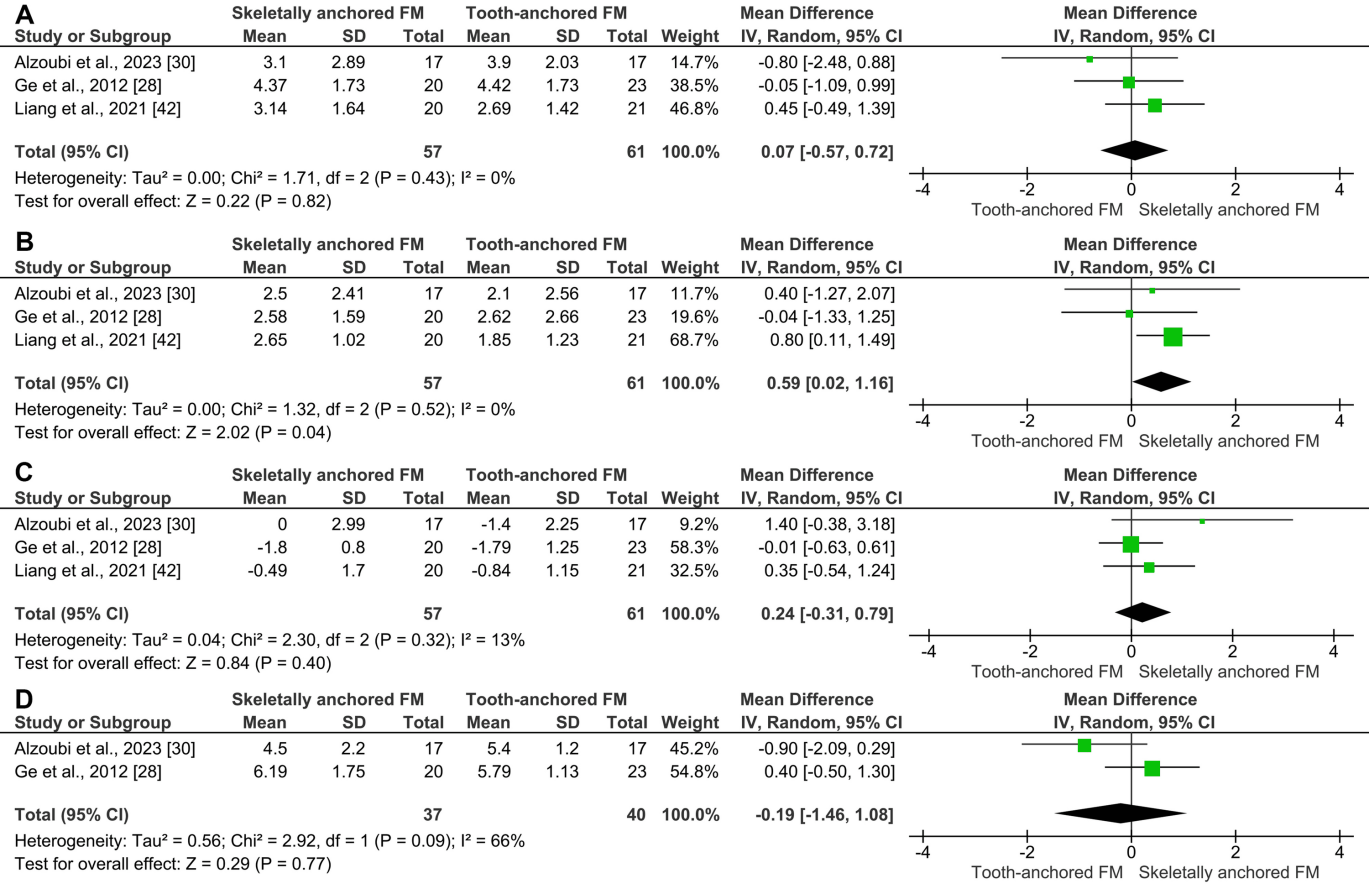
A forest plot illustrating the changes produced by the skeletally anchored face mask compared with the tooth-anchored face mask. A) Changes in the ANB angle; B) Changes in the SNA angle; C) Changes in the SNB angle; D) Changes in the overjet.

Extra-oral skeletally anchored appliances: Three studies evaluated the effects of extra-oral skeletally anchored appliances on the sagittal relationship between the two jaws. The meta-analysis of these studies using a random-effects model (n = 118) found no statistically significant difference in ANB angle changes between skeletally anchored and tooth-anchored face masks (MD = 0.07°; 95% CI: -0.57, 0.72; p = 0.82; χ² = 1.71; p = 0.43; I² = 0%; Figure 7A) [28,30,42]. Based on the GRADE approach, the evidence quality for this outcome was moderate. For maxillary position assessed by the SNA angle, the pooled results of three studies (n = 118) using a random-effects model showed a statistically significant intergroup difference, with more favorable outcomes in the skeletally anchored face mask group (MD = 0.59°; 95% CI: 0.02, 1.16; p = 0.04; χ² = 1.32; p = 0.52; I² = 0%; Figure 7B) [28,30,42]. Based on the GRADE approach, the evidence quality for this outcome was moderate.

In terms of mandibular position, measured by the SNB angle, the pooled estimate of three studies (n = 118) using the random-effects model showed no significant difference between skeletally anchored and tooth-anchored face masks (MD = 0.24°; 95% CI: -0.31, 0.79; p = 0.40; χ² = 2.30; p = 0.32; I² = 13%; Figure 7C) [28,30,42]. Based on the GRADE assessment, the quality of evidence for this outcome was considered moderate. Similarly, the meta-analysis of two studies (n = 77) assessing overjet changes using the random-effects model showed no significant difference between skeletally anchored and tooth-anchored face masks (MD = -0.19; 95% CI: -1.46, 1.08; p = 0.77; χ² = 2.92; p = 0.09; I² = 66%; Figure 7D) [28,30]. Based on the GRADE assessment, the quality of evidence for this outcome was considered very low.

Intra-oral skeletally anchored appliances: Two studies investigated the effectiveness of intra-oral skeletally anchored appliances. Meyns et al. reported a greater mean ANB increase with face mask-hybrid hyrax (3.36°) compared to mentoplate-hybrid hyrax (2.63°) [36]. Majanni and Hajeer observed a greater improvement with the BAIMT device (2.76°) than with the RMR device (1.24°) [38].

For maxillary position (SNA angle), Meyns et al. observed greater anterior movement with face mask-hybrid hyrax (2.48°) than with mentoplate-hybrid hyrax (1.99°) [36]. Majanni and Hajeer reported similar increases with the BAIMT device (1.20°) versus the RMR device (0.48°) [38].

Both studies reported mandibular retrusion. Meyns et al. noted mean SNB reductions of 0.86° and 0.64° for face mask and mentoplate groups, respectively [36]. Majanni and Hajeer observed decreases of 1.56° with the BAIM-T device and 0.76° with the RMR device [38].

Discussion

This systematic review and meta-analysis were designed to thoroughly assess the effectiveness of early intervention methods for treating anterior crossbite in children by integrating evidence from RCTs addressing dental, functional, and skeletal etiologies. By restricting inclusion to RCTs and focusing on growing patients aged 6-12 years, this review provides a high-level synthesis of the best available evidence to guide early orthodontic decision-making. The findings highlight that early intervention is generally effective across appliance categories, but the magnitude and nature of treatment effects vary considerably depending on appliance design, anchorage type, and underlying etiology of the crossbite.

Intra-oral non-skeletally anchored appliances produced limited to moderate improvements in sagittal skeletal relationships, with reported ANB changes ranging from 0.29° to 3.12° [13,26,27,32,39-41]. These values indicate that the correction of anterior crossbite is achievable with intra-oral appliances, although the skeletal contribution is generally modest. This finding is consistent with the biomechanical characteristics of these appliances, which deliver forces primarily through the dentition rather than directly to the basal skeletal structures. Correspondingly, changes in maxillary position were small, with SNA increases typically ranging from 0.7° to 2.0° [13,26,27,32,39-41], suggesting that true maxillary protraction was limited. Mandibular positional changes showed greater variability, with SNB reductions reaching up to 1.85°; however, Miamoto et al. observed a slight increase in the SNB angle. This may be because the anterior crossbite was dental, and the appliance used has no skeletal effects [26]. Despite the limited skeletal effects, intra-oral non-skeletally anchored appliances consistently produced clinically meaningful overjet correction. Reported mean increases in overjet ranged from approximately 1.4 mm to 5.9 mm [13,26,27,31,34,39,40], with the largest correction (a mean of 5.87 mm) observed with the LCP-IMT appliance [40]. The magnitude of dental correction relative to skeletal change suggests that most of these appliances primarily act through dentoalveolar mechanisms, including incisor proclination, occlusal disengagement, and removal of anterior occlusal interferences. This pattern supports the use of intra-oral appliances in dental and functional anterior crossbite and in mild skeletal discrepancies where extensive orthopedic correction is not required.

Extra-oral non-skeletally anchored appliances demonstrated a distinctly different response pattern, characterized by greater and more consistent skeletal effects. FM-RME produced a pooled ANB increase of 3.54° [32,35,39,43], with the remaining studies reporting values ranging from approximately 3.1° to 5.65° [24,25,29,33,37]. Maxillary advancement contributed substantially to this correction, as reflected by a pooled SNA increase of 1.37° [32,35,39,43], which remained significant at 1.02° following sensitivity analysis. The use of RME likely facilitated this response by enhancing the skeletal effect of protraction forces applied during growth.

Mandibular changes further contributed to the sagittal correction achieved with facemask therapy. The pooled reduction in SNB of 2.14° [32,35,39,43] decreased to 1.72° after exclusion of influential studies, indicating a backward mandibular positional change. This response may be attributed to downward and backward mandibular rotation induced by the direction of facemask forces. Overjet correction following facemask therapy ranged from 4.4 mm to 6.9 mm [25,35], and reached 8.22 mm when Class III elastics were incorporated [29], reflecting the additive dental response accompanying skeletal correction.

Although heterogeneity among facemask studies was considerable, the direction of effect was consistent across trials. Variability in force magnitude, daily wear duration, treatment length, expansion protocols, and patient compliance likely accounts for the observed differences in effect size.

When comparing skeletally anchored and tooth-anchored facemask therapy, overall sagittal correction was similar between the two approaches. The pooled MD in ANB change was only 0.07° [28,30,42], indicating that skeletal anchorage did not significantly enhance total sagittal improvement. This finding suggests that although skeletal anchorage theoretically allows more direct force transmission to the maxilla, the combined skeletal response of the maxilla and mandible remains largely unchanged. However, skeletal anchorage was associated with slightly greater maxillary advancement, as reflected by a pooled SNA difference of 0.59° [28,30,42]. This modest increase likely reflects reduced dental anchorage loss and more efficient force delivery to the maxillary skeletal structures. This difference in maxillary advancement did not result in superior mandibular or overjet outcomes, as SNB and overjet changes were comparable between anchorage types. These findings indicate that while skeletal anchorage may alter the distribution of skeletal effects, its impact on overall sagittal correction is limited. The modest advantage in SNA angle associated with skeletal anchorage may be clinically relevant in selected cases with severe maxillary deficiency or compromised dental anchorage; however, the additional surgical procedures, costs, and patient burden must be carefully weighed against the potential benefits.

Intra-oral skeletally anchored appliances showed promising outcomes in the early treatment of skeletal anterior crossbite, despite being evaluated in only two RCTs [36,38]. By transmitting orthopedic forces directly to the basal bone, these appliances limit dentoalveolar compensation and allow greater expression of skeletal effects. BAIMT [38] produced greater sagittal correction than the RMR, with a mean ANB increase of 2.76° compared with 1.24°. This superior effect was mainly driven by enhanced mandibular restraint, reflected by a larger SNB reduction (1.56° vs. 0.76°), likely due to improved control of mandibular position through skeletal anchorage, along with modest maxillary advancement (SNA increase: 1.20° vs. 0.48°). Similarly, Meyns et al. utilized the Alt-RAMEC protocol together with a mentoplate-Hyrax hybrid appliance for anteroposterior traction. This approach was compared with a face mask-Hyrax hybrid system, and ANB increases of 2.63° and 3.36° were reported for the respective groups. The face mask-hybrid Hyrax favored maxillary advancement (SNA: 2.48° vs. 1.99°), whereas the mentoplate-hybrid Hyrax protocol emphasized mandibular restraint (SNB reduction: 0.86° vs. 0.64°), reflecting differences in force application and anchorage location.

Limitations of the current work

A number of limitations and considerations should be acknowledged when interpreting the results of this systematic review. First, although 21 RCTs were included in the review, the quality of individual study methodology was highly variable, and several were assessed as high risk of bias. This variability in study quality reduces confidence in the pooled estimates. Second, considerable clinical and statistical heterogeneity was present across studies. This was particularly evident in the trials evaluating facemask therapy, where variations in appliance design, force magnitude, duration of daily wear, treatment protocols, and patient compliance likely contributed to this heterogeneity. Third, the majority of the included articles focused on skeletal anterior crossbite, with only three trials considering dental and functional etiologies. This imbalance limits the generalizability of findings across all types of anterior crossbite. Finally, the outcome measures used were not consistent across all trials; some relied on cephalometric radiographs, and others used dental models or CBCT images. This variability may influence the comparability of results.

## Conclusions

The findings of this review support early intervention for growing patients with anterior crossbite, with treatment choice guided by the underlying cause and desired outcomes. Intra-oral, non-skeletally anchored appliances are effective for dentoalveolar correction and are well-suited for mild skeletal discrepancies, as well as dental and functional anterior crossbites. They are particularly useful when minimal skeletal change is needed or when compliance with extra-oral devices is expected to be low. In contrast, facemask therapy remains the most reliable option for achieving significant skeletal correction, especially when combined with rapid maxillary expansion, although skeletal anchorage may not always be necessary due to similar overall sagittal outcomes.

From a research perspective, the review highlights the need for higher-quality evidence. Current findings, based on studies with very low to moderate certainty, should be interpreted cautiously. Well-designed RCTs and advanced methods such as network meta-analysis are needed to strengthen the evidence base and guide clinical practice.

## Appendices

Appendix 1

**Table 6:** Electronic search strategy used in the current systematic review.

Electronic database	Search strategy
PubMed	#1 ("Malocclusion, Angle Class III" OR "Class III malocclusion" OR "skeletal Class III" OR "mandibular prognathism" OR "maxillary retrusion" OR "mandibular protrusion" OR "skeletal anterior crossbite" OR "functional anterior crossbite" OR "dental anterior crossbite") #2 ("Dentition, Mixed" OR "mixed dentition" OR "early treatment" OR "interceptive" OR "child" OR "children" OR "juvenile") #3 ("skeletal changes" OR "dentoalveolar changes" OR "upper incisor inclination" OR "lower incisor inclination" OR "overjet" OR "overbite" OR "treatment duration" OR "soft tissue changes") #4 ("Orthodontic Appliances" OR "Orthodontic Appliances, Functional" OR "removable orthodontic appliance" OR "functional appliance" OR "facemask" OR "chin cup" OR "Activator III" OR "Bionator III" OR "reverse pull headgear" OR "Frankel III" OR "reverse twin block" OR "removable mandibular retractor" OR "tandem appliance" OR "skeletal anchorage" OR "bone anchored maxillary protraction" OR "Orthodontic Anchorage Procedures") #1 AND #2 AND #3 AND #4
CENTRAL (The Cochrane Library)	#1 ("Malocclusion, Angle Class III" OR "Class III malocclusion" OR "skeletal Class III" OR "mandibular prognathism" OR "maxillary retrusion" OR "mandibular protrusion" OR "skeletal anterior crossbite" OR "functional anterior crossbite" OR "dental anterior crossbite") #2 ("Dentition, Mixed" OR "mixed dentition" OR "early treatment" OR "interceptive" OR "child" OR "children" OR "juvenile") #3 ("skeletal changes" OR "dentoalveolar changes" OR "upper incisor inclination" OR "lower incisor inclination" OR "overjet" OR "overbite" OR "treatment duration" OR "soft tissue changes") #4 ("Orthodontic Appliances" OR "Orthodontic Appliances, Functional" OR "removable orthodontic appliance" OR "functional appliance" OR "facemask" OR "chin cup" OR "Activator III" OR "Bionator III" OR "reverse pull headgear" OR "Frankel III" OR "reverse twin block" OR "removable mandibular retractor" OR "tandem appliance" OR "skeletal anchorage" OR "bone anchored maxillary protraction" OR "Orthodontic Anchorage Procedures") #1 AND #2 AND #3 AND #4
Scopus	#1 TITLE ABS-KEY= ("Malocclusion, Angle Class III" OR "Class III malocclusion" OR "skeletal Class III" OR "mandibular prognathism" OR "maxillary retrusion" OR "mandibular protrusion" OR "skeletal anterior crossbite" OR "functional anterior crossbite" OR "dental anterior crossbite") #2 TITLE ABS-KEY= ("Dentition, Mixed" OR "mixed dentition" OR "early treatment" OR "interceptive" OR "child" OR "children" OR "juvenile") #3 TITLE ABS-KEY= ("skeletal changes" OR "dentoalveolar changes" OR "upper incisor inclination" OR "lower incisor inclination" OR "overjet" OR "overbite" OR "treatment duration" OR "soft tissue changes") #4 TITLE ABS-KEY= ("Orthodontic Appliances" OR "Orthodontic Appliances, Functional" OR "removable orthodontic appliance" OR "functional appliance" OR "facemask" OR "chin cup" OR "Activator III" OR "Bionator III" OR "reverse pull headgear" OR "Frankel III" OR "reverse twin block" OR "removable mandibular retractor" OR "tandem appliance" OR "skeletal anchorage" OR "bone anchored maxillary protraction" OR "Orthodontic Anchorage Procedures") #1 AND #2 AND #3 AND #4
Web of Science	#1TS= ("Malocclusion, Angle Class III" OR "Class III malocclusion" OR "skeletal Class III" OR "mandibular prognathism" OR "maxillary retrusion" OR "mandibular protrusion" OR "skeletal anterior crossbite" OR "functional anterior crossbite" OR "dental anterior crossbite") #2TS= ("Dentition, Mixed" OR "mixed dentition" OR "early treatment" OR "interceptive" OR "child" OR "children" OR "juvenile") #3TS= ("skeletal changes" OR "dentoalveolar changes" OR "upper incisor inclination" OR "lower incisor inclination" OR "overjet" OR "overbite" OR "treatment duration" OR "soft tissue changes") #4TS= ("Orthodontic Appliances" OR "Orthodontic Appliances, Functional" OR "removable orthodontic appliance" OR "functional appliance" OR "facemask" OR "chin cup" OR "Activator III" OR "Bionator III" OR "reverse pull headgear" OR "Frankel III" OR "reverse twin block" OR "removable mandibular retractor" OR "tandem appliance" OR "skeletal anchorage" OR "bone anchored maxillary protraction" OR "Orthodontic Anchorage Procedures") #1 AND #2 AND #3 AND #4
Google Scholar	("Malocclusion, Angle Class III" OR "Class III malocclusion" OR "skeletal Class III" OR "mandibular prognathism" OR "maxillary retrusion" OR "mandibular protrusion" OR "skeletal anterior crossbite" OR "functional anterior crossbite" OR "dental anterior crossbite") AND ("Dentition, Mixed" OR "mixed dentition" OR "early treatment" OR "interceptive" OR "child" OR "children" OR "juvenile") AND ("skeletal changes" OR "dentoalveolar changes" OR "upper incisor inclination" OR "lower incisor inclination" OR "overjet" OR "overbite" OR "treatment duration" OR "soft tissue changes") AND ("Orthodontic Appliances" OR "Orthodontic Appliances, Functional" OR "removable orthodontic appliance" OR "functional appliance" OR "facemask" OR "chin cup" OR "Activator III" OR "Bionator III" OR "reverse pull headgear" OR "Frankel III" OR "reverse twin block" OR "removable mandibular retractor" OR "tandem appliance" OR "skeletal anchorage" OR "bone anchored maxillary protraction" OR "Orthodontic Anchorage Procedures")
